# Study of Fecal and Urinary Metabolite Perturbations Induced by Chronic Ethanol Treatment in Mice by UHPLC-MS/MS Targeted Profiling

**DOI:** 10.3390/metabo9100232

**Published:** 2019-10-16

**Authors:** Olga Deda, Christina Virgiliou, Amvrosios Orfanidis, Helen G. Gika

**Affiliations:** 1 Department of Medicine, Aristotle University of Thessaloniki, 54124 Thessaloniki, Greece; 2 Center for Interdisciplinary Research of the Aristotle University of Thessaloniki (KEDEK), 57001 Thessaloniki, Greece; 3 Department of Chemistry, Aristotle University of Thessaloniki, 54124 Thessaloniki, Greece

**Keywords:** ethanol, alcohol, toxicity, metabolomics, feces, urine, ALD, mice

## Abstract

Alcoholic liver disease (ALD) as a consequence of ethanol chronic consumption could lead to hepatic cirrhosis that is linked to high morbidity and mortality. Disease diagnosis is still very challenging and usually clear findings are obtained in the later stage of ALD. The profound effect of ethanol on metabolism can be depicted using metabolomics; thus, the discovery of novel biomarkers could shed light on the initiation and the progression of the ALD, serving diagnostic purposes. In the present study, Hydrophilic Interaction Liquid Chromatography tandem Mass Spectrometry HILIC-MS/MS based metabolomics analyisis of urine and fecal samples of C57BL/6 mice of both sexes at two sampling time points was performed, monitoring the effect of eight-week ethanol consumption. The altered hepatic metabolism caused by ethanol consumption induces extensive biochemical perturbations and changes in gut microbiota population on a great scale. Fecal samples were proven to be a suitable specimen for studying ALD since it was more vulnerable to the metabolic changes in comparison to urine samples. The metabolome of male mice was affected on a greater scale than the female metabolome due to ethanol exposure. Precursor small molecules of essential pathways of energy production responded to ethanol exposure. A meaningful correlation between the two studied specimens demonstrated the impact of ethanol in endogenous and symbiome metabolism.

## 1. Introduction

Chronic consumption of ethanol is a leading cause of alcohol liver disease (ALD). As this implication is the far end of intermediate and reversible stages characterized by hepatic steatosis, which is caused by lipid metabolism dysregulation mechanisms triggered by ethanol, early diagnosis of detrimental ethanol consumption is of high significance. Unfortunately, the diagnosis of ALD can be clinically challenging as there is no single test that confirms the presence of the disease and patients may not always provide clear information concerning their alcohol consumption patterns. Also, clinical findings may be absent or negligible in early ALD, such as simple steatosis and mild steatohepatitis, which generally have no obvious clinical symptoms [1].

Typical diagnostic tests would include serum transaminases and aspartate transaminase (AST) or in unclear cases, liver imaging and biopsy. Thus, the development of noninvasive tests based on metabolic markers indicative of early stage ALD could augment the power of regular tests, improving the potential for early detection of ethanol toxic effects and promoting measures toward disease prevention [2,3].

Metabolomics can provide insight about the changes in small-molecule intermediary products (i.e., metabolites) of the organism exposed to drugs or other stimuli and has been proven to be a powerful tool for biomarker discovery [4,5]. Undoubtedly, alcohol consumption has a great impact on the metabolome, inducing a biochemical reaction cascade. In addition to the highly affected lipid metabolism, alcohol intake leads to profound effects in other essential biochemical pathways [6]. Metabolomics can therefore promote the understanding of the profound metabolic perturbations induced by ethanol consumption which finally lead to liver inflammation and necrosis. Hence, it can pave the way from early diagnosis to prognosis and monitoring the progression of ALD.

Ethanol-related alterations that act as a trigger to induce associated diseases have been mainly found in organs that are recognized as the targets of ethanol toxicity, such as the liver and brain. However, the key aim of the biomarker discovery field is the use of easily accessible and noninvasive samples such as urine and fecal samples. Several metabolic profiling studies have been conducted for ethanol toxicity in various sample matrices [7] such as the liver [8,9], brain [9], blood [10], urine [11,12,13] or fecal samples [13,14] in patients and especially in animal models. The preferred biological matrix in these studies is mainly the blood; however, urine, as well as fecal samples (especially in combination) can provide a very informative picture of the organism’s physiology in a noninvasive way. Moreover, fecal samples can offer the potential of revealing ethanol toxicity effects in the very early stages, as ethanol-induced gut microbiota alterations are able to affect the whole organism through their signaling molecules. It has been shown that ethanol consumption can lead to a leaky gut barrier and dysbiosis, which is associated with ALD manifestation [15,16]. Fecal metabolites are the products of the microbiome, some of which are absorbed into the circulation and eventually, after being metabolized by the host (co-metabolism), they are excreted in urine. Due to this, a complementary picture of the fecal and urine metabolome can provide new knowledge on the mechanisms involved and the role of the gut microbiome–host interaction in ALD.

Interesting findings from metabolic profiling studies on urine or fecal samples have been published. Gao et al. [17] have reported a different impact on the urine metabolome between different strains of rats under ethanol treatment, namely Sprague–Dawley (SD) and Wistar, while NMR-based metabolomics analysis of C57BL/6J urine samples have revealed taurine depletion and an excess of lactate, n-acetylglutamine and n-acetylglycine [8]. Shi et al. [18] have suggested N-acetyltaurine as a urine biomarker of ethanol toxicity (which is derived from taurine and acetate abundance). However, reports of ethanol impact on the fecal metabolome are limited; only recently there have been studies that show alterations of the microbiome and metabolome of the gut [3,6,14,19,20,21,22,23]. Xie et al. [6] analyzed the content of the gastrointestinal (GI) tract from stomach to rectum of SD rats exposed to ethanol for eight weeks, indicating the profound impact of ethanol. They used Ultra-high performance liquid chromatography tandem mass spectrometry UHPLC-MS analysis of volunteered and enforced ethanol drinking BALC/c mice [24], elucidating perturbations of bile acid metabolism and neurotransmitter metabolism, while histopathological analysis demonstrated liver and colon damage. The latter findings confirm that the liver, gut and brain are metabolically connected to each other, as gut microbiota-associated metabolites transfer positive or negative messages that are even able to cross the blood–brain barrier, explaining why gut microbiome attract scientific interest.

In the present study, targeted metabolic profiling of urine and fecal samples from ethanol dependent C57BL/6 mice of both sexes was carried out for two time (sampling) points. The study attempts to enrich our limited knowledge on fecal samples, compared to results of other matrices [3,6,7,14,22]. The interaction and cross-talk effects between the liver and gut is a hot topic of investigation, as recent studies have clarified the important role played by the gut symbiome in mammalian health maintenance [25]. Correlation of the findings in the two samples can provide information on the metabolism involved in ALD and can offer a combinatorial metabolic fingerprint that is valuable for early diagnosis. Further to this, although the differences in ethanol metabolism between the two sexes in mammals are well-known [26,27,28,29,30], this is to the best of our knowledge the first metabolomics-based study investigating the metabolic impact of ethanol consumption in the two sexes.

## 2. Results

This study aimed to investigate the effect of ethanol on the metabolic content of urine and fecal samples in a mouse model simulating chronic exposure to ethanol. The intention was to highlight patterns of alteration in the metabolic phenotype of these samples indicative of a toxic ethanol effect. The applied protocol lasted for a period of eight weeks, however, urine and fecal samples presented in this work were collected up until the 20th day of exposure. At the end of the experiment, based on the histopathology findings, it was concluded that ethanol treatment induced mild steatohepatitis, but no liver fibrosis. Thus, it could be considered that the protocol may provide early stage biomarkers of steatohepatitis in the first period of the experiment, where the two sampling points were performed. This is of high importance, as the metabolic phenotype in the early stage of a reversible implication caused by the hepatotoxic effect of ethanol seems even more meaningful.

Monitoring the health condition of mice during the experiment revealed an average weight loss of 15% and 10% for male and female animals, respectively. In addition, high mortality rates were observed in the female population mainly the first 10 days; thus, a smaller number of samples could be collected. The higher susceptibility of females to ethanol is well documented [31,32,33,34]. It has been attributed to the increased activation of Kupffer cells via enhanced CD14 expression, which activates NF-κB, leading to an increase in TNF-α mRNA expression in the liver and thus a more severe and rapidly developing liver injury in female mice. Our observation can therefore be explained by existing data and provides awareness of the different levels of disease progression between the two genders.

The analysis of samples by the applied method provided the detection of 80 and 66 low molecular weight metabolites in urine and fecal samples, respectively. These comprised amino acids, carbohydrates, organic acids, vitamins, nucleotides and amines. Data analysis was based on peak areas and included data quality assessment with the implementation of a Quality Control QC sample. QC data indicated precise analytical measurements.

**Figure 1:**
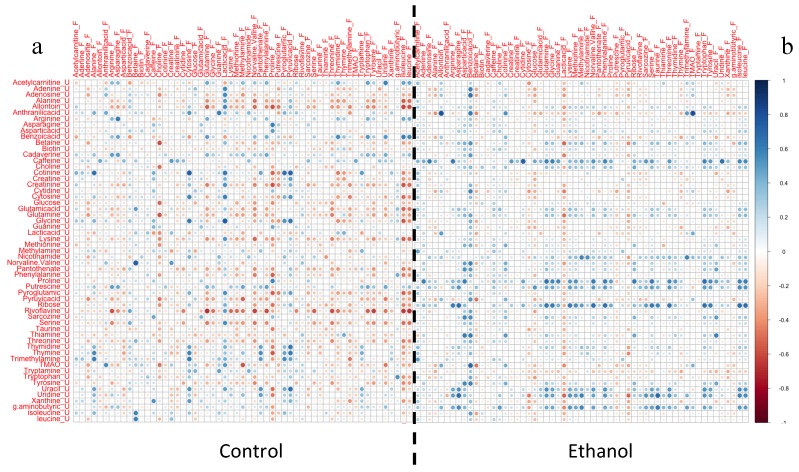
Pearson correlation heatmaps for the commonly detected metabolites in urine (U) and fecal (F) samples exhibiting the correlation pattern between the urinary and the fecal metabolome in (**a**) the control mice and in (**b**) the ethanol treated mice.

A comparative study between urine and fecal samples of the same animals indicated in total 60 common metabolites in the two matrices (data are provided in Supplementary Figure S1). The commonly detected metabolites were correlated between the urine and fecal samples for the control mice and for those undergoing ethanol treatment. Pearson correlation heatmaps, as shown in Figure 1a,b, indicate alterations in the correlation patterns between the two specimens after treatment with ethanol, suggesting that ethanol interferes in the balance of gut microbial metabolism and host interaction. The linear relationship between metabolites of urine and fecal samples is disrupted or in extreme cases, their correlation is reversed with ethanol treatment. As an example, fecal choline and lactate have a positive correlation with all urinal metabolites in control mice, while they have a negative correlation in ethanol treated mice. Similar findings are also observed for other key molecules such as pyruvate and glucose, highlighting the strong metabolic impact of ethanol exposure.

**Figure 2:**
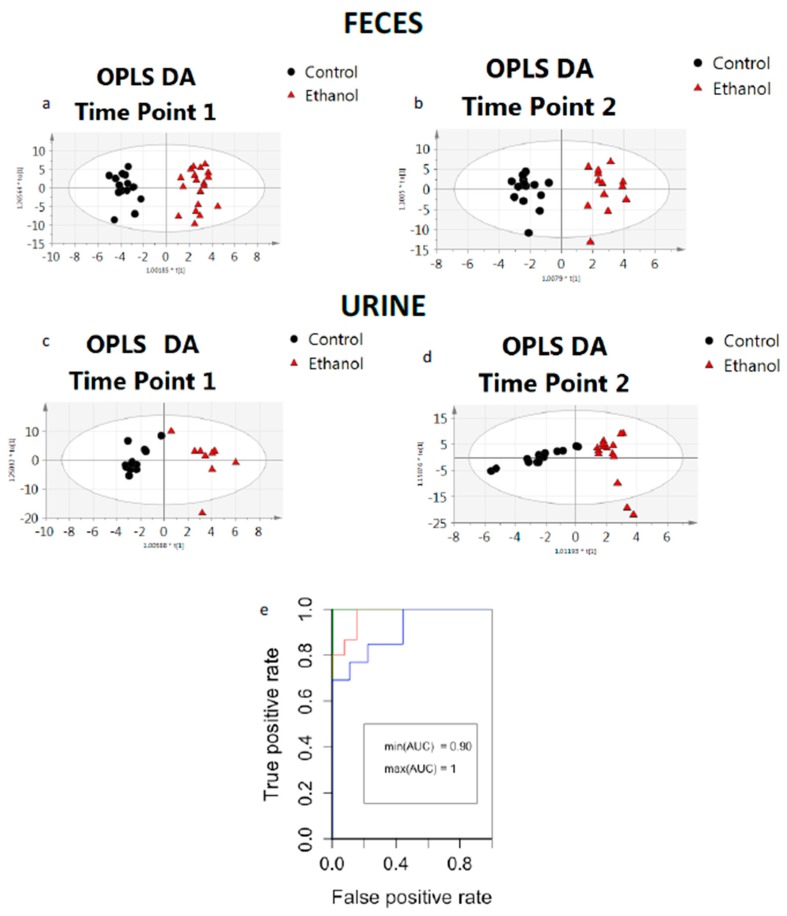
OPLS DA score plots constructed based on metabolic profiles acquired from fecal samples at (**a**) sampling time point 1 and (**b**) time point 2 and the corresponding score plots constructed based on metabolic profiles of urine samples at (**c**) sampling time point 1 and (**d**) time point 2, (*x*-axis: first principal component to [1]; *y*-axis: the first component to [1]), while in (**e**) overlay ROC curves for the models are shown.

Based on multivariate statistical analysis, alterations in the hydrophilic metabolite content after ethanol treatment was exhibited. Orthogonal Partial Least Square-Discriminant Analysis OPLS DA score plots indicated distinct differences between samples from control and ethanol treated mice. The differentiation of metabolite content with ethanol treatment was observed in both matrices and for both time points. The score plots of OPLS DA analysis are presented in Figure 2a,b for fecal samples at the two time points and in Figure 2c,d for urine samples at the two time points. The *p* value of cross validated ANOVA analysis (CV analysis) was <0.05 in most cases, indicating the statistical significance of the investigated models (with the exception of urine samples at the first time point, where the *p* value was slightly increased (*p* = 0.09)). Further statistical analysis was also performed for this dataset. Statistics from all models are summarized in Table 1. From these data, it can be concluded that the effect of ethanol on the fecal metabolome was more profound when compared to the urine metabolome, and this was even more noticeable in male mice.

Furthermore, Receiver Operating Characteristic ROC curves of the constructed models for the urine and fecal samples provided an indication of the robustness of the models with calculated Area Under the Curve AUC values ranging from 0.90 for urine samples at the first time point to 1.0 for fecal samples at the second time point. Overlay ROC curves for the models are illustrated in Figure 2e.

Data from the multivariate and univariate analysis demonstrated the ability of these modeling procedures to distinguish the two groups of mice (control vs. ethanol treated) based on the dysregulated metabolites. Several metabolites that were significantly affected by ethanol treatment based on the criterion of *p* < 0.05 were revealed by a two-paired t-test. More specifically, in the fecal metabolic profile, 19 metabolites were found to be altered in ethanol treated mice samples at the first time point, from which 15 were continued to be altered in the second time point. However, different trends were observed over the two sampling periods for the 15 metabolites that continued to be altered. For some metabolites (e.g., amino acids), an increasing trend was observed, while for others (e.g., amines), a decrease was observed in the earlier time point followed by an increase in the second time point. At the second time point, 16 additional metabolites were also found to be dysregulated in ethanol treated mice. The metabolites and their trends, as well as the metabolic pathways that are involved, are summarized in Supplementary Table S2.

**Figure 3:**
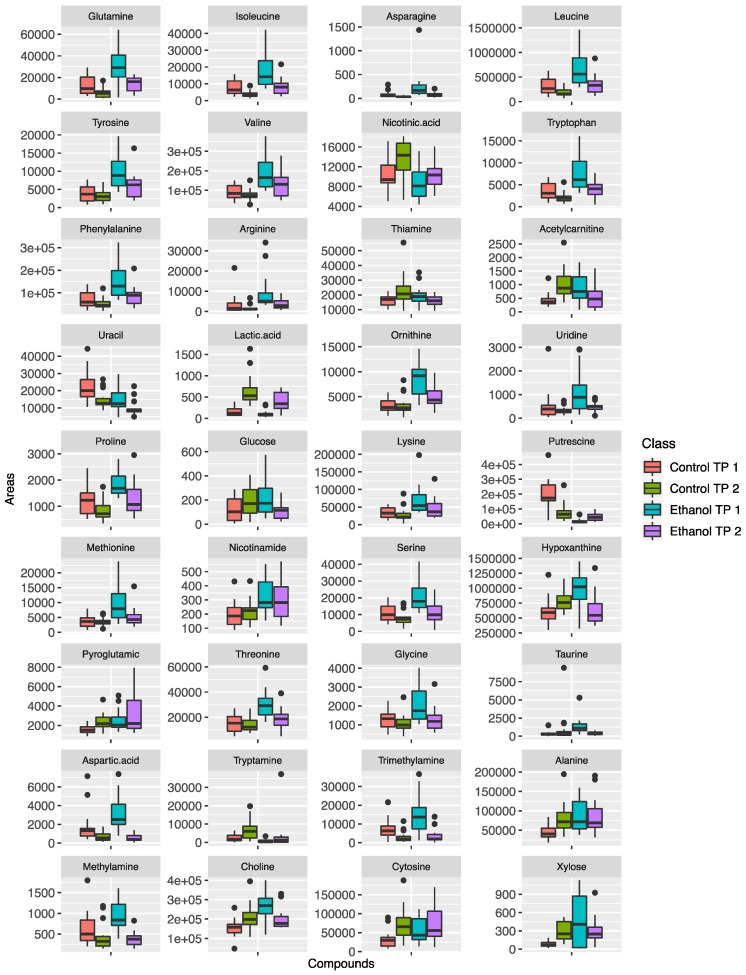
Box plots for the metabolites that were found to differentiate significantly in the fecal samples from ethanol treated mice.

**Figure 4:**
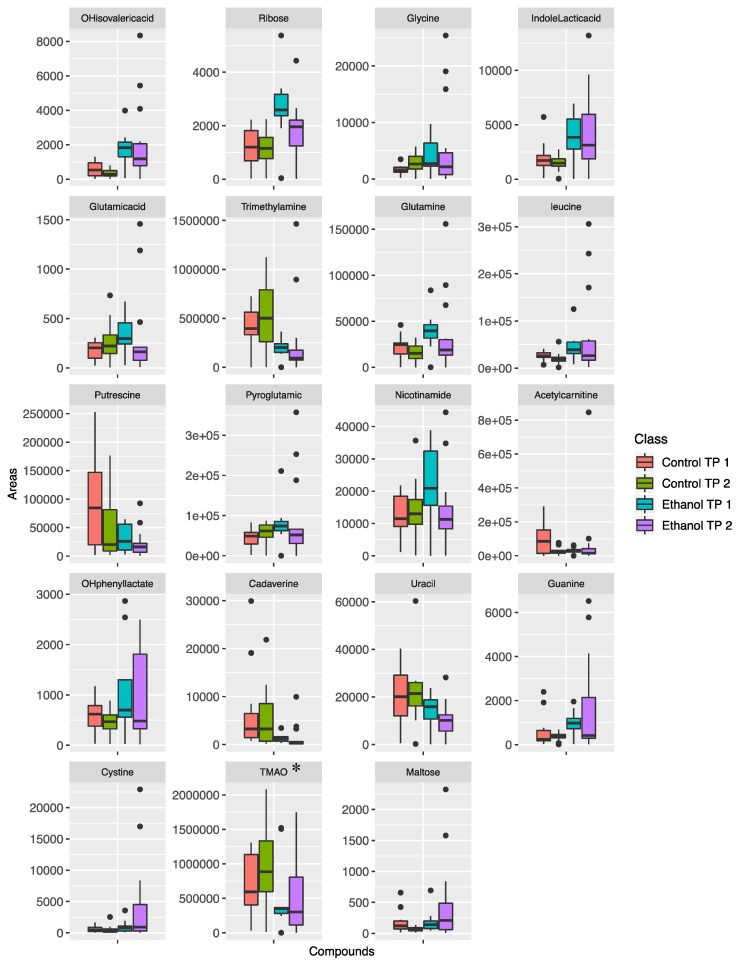
Box plots for the metabolites that were found to differentiate significantly in the urine samples of ethanol treated mice. * Trimethylamine-N-oxide.

As mentioned above, by comparing fecal and urine samples, it was observed that the metabolic profile of urine samples was less affected by ethanol treatment. More precisely, in urine, 12 metabolites were found to be significantly changed at the first time point and 13 metabolites were found to be significantly different at the second time point. From the 12 metabolites altered in the first time point, half of them continued to be altered in the second time point, as shown in Supplementary Table S3. The majority of these metabolites show an increasing trend from the first time point to the second time point, with the exception of trimethylamine. Graphically, the trends of the metabolites that were found significantly altered are shown as box plots in Figure 3 for fecal samples and in Figure 4 for urine samples.

With regard to the impact of ethanol on the urine and fecal metabolome in relation to sex, differences can be observed. Multivariate statistical analysis of samples collected at the first time point showed significant differentiations between control and ethanol treated male mice in both matrices, while for female mice, this was observed only in fecal samples. At the second time point, control and ethanol treated male mice preserved their differences based only on the fecal samples, while female mice showed statistically significant differences in urine. OPLS DA score plots of these models are provided in Supplementary Figure S2, whereas statistical data of the constructed OPLS DA models are included in Table 1. Altered metabolites with *p* < 0.05 of the corresponding male and female models are presented as bar plots of log2 fold change in Supplementary Figure S3. The metabolites found to be altered in male were not the same as those that were altered in female mice.

## 3. Discussion

Alcoholic liver disease (ALD) remains an issue of great concern since it is intensely associated with high morbidity and mortality through induced hepatic cirrhosis triggered by chronic ethanol consumption [35]. State-of-the-art technologies provide the ability to carry out a detailed and reliable metabolic fingerprinting that is able to find the metabolic traces of the impact of ethanol and the induced hepatotoxicity [7].

The applied targeted method managed to reveal ethanol derived metabolic changes in both analyzed biological specimens, with the strongest impact on the fecal metabolome in comparison to urine. The effects of ethanol exposure on male mice demonstrated a greater impact in contrast to female mice, as evidenced by the altered metabolites. On the other hand, despite the smaller number of differentiated metabolites, female mice were more vulnerable to ethanol consumption, since many losses were observed in the female population. The evaluation of the fecal metabolome resulted in differences for both sexes and time points. The major factors responsible for the differentiations are the sex-dependent mammalian metabolism of ethanol and the gut microbiome population.

The selection of an experimental animal model was of great concern. Previous publications were a determining factor for the selection of the mice strain [36] regarding volunteered ethanol consumption. However, the implementation of the study elucidates some limitations that are unforeseeable in pilot experiments related to the small sample size, the vulnerability of mice and difficulties in developing steatosis.

The fecal metabolome of ethanol treated mice was dramatically changed compared to control mice. Metabolic perturbations derived from ethanol consumption were observed at a great scale for both studied time points. It is remarkable that in the first time point, 12 out of 66 differentiated metabolites were increased. In contrast, only seven metabolites were found to be decreased. Interestingly, in the second time point, 29 and two differentiated metabolites were also found to be increased and decreased, respectively. From the altered metabolites in the second time point, 15 were already altered in the first time point, while L-acetylcarnitine, putrescine and tryptamine demonstrated an adverse trend between the two time points. It could be hypothesized that the adverse trend reflects a progressively enhanced metabolic stress derived from prolonged ethanol exposure and the inability and difficulty of the mice to overcome such a severe metabolic imbalance. Similar adverse trends have been observed in the past by our group in a similar study comparing blood and urine, where samples were collected in the second and fourth week of exposure [37] 

The biochemical pathways of major precursors that are essential for energy generation were significantly affected by ethanol consumption. Nitrogen metabolism, ammonia recycling and the urea cycle, which are fundamental processes for cellular nitrogen utilization, were found to be perturbed at both sampling time points. The liver, the main target of ethanol toxicity, is responsible for the utilization of amino acids to induce protein synthesis, pyrimidine and purine synthesis, ketogenesis, carbohydrate formation and de novo synthesis of non-essential amino acids [38]. 

Precursor molecules related to the aforementioned processes such as cytosine, hypoxanthine, thiamine, uracil and uridine were significantly altered, and their perturbed values are associated with a hepatic inflammatory condition [39,40].

A noticeable impact of ethanol consumption on amino acid metabolism was observed, since the metabolism of alanine–aspartate–glutamate, glycine–serine–threonine, valine–leucine–isoleucine, phenylalanine and tyrosine were disturbed.

A characteristic reduction in amino acid levels follows chronic ethanol consumption due to enhanced liver blood protein synthesis. Protein accumulation, in parallel with lipids, leads to water influx which consequently affects the hepatic architecture.

Severe hepatic damage, as a consequence of progressive alcohol consumption, could result in cirrhotic conditions accompanied with aromatic amino acids and methionine increase and glutathione decrease in blood [41]. Fixing the widespread dysmetabolism of hepatic cells caused by alcohol consumption is not an easy task due to the absence of major cellular feedback mechanisms [42]. However, in the present study, the observed increase in some amino acid levels (phenylalanine, tyrosine, valine, leucine) can be attributed to the perturbation of gut microbiota populations. The metabolic connection of the gut and the ALD suffering liver through the circulation [22] explains the qualitative and quantitative effect of ethanol intake on the gut microbiome, described by “dysbiosis” and bacterial overgrowth, respectively [43]. Both physiological and metabolic alteration of ethanol induced gut microbiota is reflected in the fecal metabolome. The statistically significant metabolites and the trend of their behavior are due to the combined effect of the mammalian gut microbiome and their symbiome metabolic alterations. The exact mechanism of ethanol induced symbiotic dysmetabolism is still unspecified [22]. For example, lysine could follow two fates; either ammonia production or supplying the route from acetyl-CoA to butyric acid.

Among the hallmarks of ethanol toxicity in hepatic cells is the inhibition of glycolysis and gluconeogenesis, the Krebs cycle, fatty acid oxidation and the induction of ketogenesis due to the remarkable reduction of NAD–NADH via ethanol dehydrogenase activity [44]. Ethanol presents the unique ability among other toxins to change almost all biochemical processes of hepatic lipid metabolism. The ethanol derived lipid over-accumulation induces liver steatosis with mild inflammation in long-term consumption [45]. Altered β-oxidation was also evident by the effected acetyl-L-carnitine. Molecules that supply the Krebs cycle were disturbed by ethanol consumption, altering the metabolism towards an enhanced production of lactic acid, causing lactic acidosis and secondly ketogenesis, as evidenced by lactic acid alterations. Long-term ethanol consumption is also very harmful for mitochondria, leading to the consequent inhibition of electron transport and oxidative phosphorylation [21]. As ethanol is metabolized, the formation of acetaldehyde and free radicals is inevitable. The ingested non-metabolized ethanol irreversibly damages the liver via inflammation triggered mechanisms [46,47,48].

In a very interesting study, decreased levels of glucose, lactate, and alanine were seen in rat livers and serum, while increased levels of liver and serum acetate and serum acetoacetate were found regardless of the ethanol exposure dose. The authors concluded that the depletion of alanine, acting with feedback-inhibition regulation activated pyruvate kinase, induced a perpetual cycle that removes phosphoenolpyruvate from gluconeogenesis, explaining the “empty calorie” phenomenon of ethanol [9].

In contrary to previous findings, Bradford et al. [8] observed elevated hepatic glucose and lactate levels in mice that were attributed to increased glycolysis and hypoxic effects, respectively. They also found that the levels of tyrosine and precursor molecules associated with blood coagulation factors were elevated.

Upregulation of lactic acid and downregulation of purine catabolism attributed to an unbalanced hepatic redox system triggered by ethanol-induced NAD+ depletion were found using NMR-based metabolomics of acute ethanol exposure in the volunteer population [11].

Hepatic nonpolar metabolites of ethanol exposed rats and mice such as fatty acyls/acids/ethyl esters, glycerolipids and phosphatidylethanol homologues were extracted using high mass accuracy multi stage Mass Spectrometry MSn analysis and evidenced in the extensive metabolic response to ethanol. Remarkably, unique markers were revealed for both species, underlying the metabolic differences [49] in the disturbed lipid metabolism.

In the present study, mice urine samples demonstrated milder metabolic changes compared to the extracted fecal metabolic alterations. In the analyzed urine samples, 12 and 13 metabolites were found altered in the first and second sampling time point, respectively. From the above differentiated metabolites, six were found to be in common, namely hydroxyphenyllactic acid, indolelactic acid, D-ribose, (S)-3 hydroxyisobutyric acid and L-isoleucine/L-isoleucine. They presented with similar trends for both time points. Trimethylamine was the only exception that presented with adverse effects between the two time points. Biochemical pathways mainly associated with the metabolism of amino acids and their derivatives, carbohydrates, fatty acids, and nucleotides were changed in response to ethanol consumption. Furthermore, in accordance with the related studies, previously demonstrated ethanol consumption biomarkers were confirmed based on the obtained results. Hydroxyphenyllactic acid and indolelactic acid, related to tyrosine and tryptophan metabolism, are considered to be metabolomics-based hallmarks of ethanol consumption. Chronic ethanol exposure leads to significant upregulation of tryptophan metabolism, while tyrosine, phenylalanine and lysine metabolism were also affected, as demonstrated in similar studies [2,8,50]. Tryptophan, phenylalanine and tyrosine metabolism were also altered on a great scale in analyzed fecal samples from the ethanol treated mice. These specific biochemical pathways dominate in the gut microbiome metabolome. Basic precursors are metabolized by the bacteria in order to fully cover the energetic demands for intestinal epithelium mucosal maintenance and the development of the microbial community. The exact origin of the alterations of urine metabolites is difficult to attribute to either host or symbiome metabolism due to the similarity of the biochemical routes and only speculations can be made.

Overall, more metabolites were observed from fecal metabolome profiling with an adverse trend over the period of exposure, compared to urine. Obviously, the reason for this is the strong effect of ethanol toxicity on the gut microbiome, while the urine metabolome responded primarily to the decline in energy homeostasis triggered by the strong stimulus of chronic ethanol exposure. All the precursors involved in each related biochemical pathway should be monitored in order to precisely explain the direction of the metabolism of the significantly altered metabolites. At a more accurate level, biochemical interpretation should take into account the hepatic enzymatic activity of ethanol, which is also dependent on specific factors (e.g., sex) and how this could reflect in the fecal and urine metabolome. In addition, cross-talk effect phenomena should be considered when the biochemical fate of key molecules is investigated.

Finally, it should be noted that the present study expands the already confirmed diagnostic panel of biomarkers proposing that the combination of the two studied matrices could enhance the diagnostic value. Metabolites found to be perturbed in both specimens could constitute biomarkers with high specificity.

## 4. Materials and Methods

### 4.1. Chemicals and Reagents

The solvents acetonitrile and methanol of LC/MS grade as well as ammonium formate and formic acid (mobile phase additives) were purchased from Sigma Aldrich, St. Louis, MO, USA and Merck, Darmstadt, Germany. Ultrapure Water (18.2 MΩ cm) was obtained from a Milli-Q purification system (Merck, Darmstadt, Germany). Saline solution (NaCl 0.9%) was purchased from VIOSER S.A.

### 4.2. In vivo Study

The chronic ethanol exposure in vivo experiment was performed for a period of 8 weeks in the Animal Physiology Facility of the Veterinary Medicine School of Aristotle University of Thessaloniki with 38 C57BL/6 mice at 8–10 weeks of age. Fecal and urine samples were collected on the same days at two time points (10 days and 20 days from the beginning of ethanol exposure) and were immediately frozen. At the end of the experiment, animals were sacrificed by cervical dislocation and tissues were collected for further studies.

Animals of both sexes, 21 males and 17 females, were divided in two groups and treated with either an ethanol liquid diet or control diet (11 males ethanol-treated vs. 10 controls and 9 females ethanol-treated vs. 8 controls). The protocol by Bertola et al. [1] was adapted and the animals were fed ad libitum with the Lieber–DeCarli ethanol diet, containing 5% extra pure ethanol. Caloric intake for ethanol treated mice and controls was balanced by adding an appropriate amount of maltose dextrin to the control diet. An acclimatization period to the liquid diet was carried out for 7 days. Preparation of the liquid diet was performed daily in accordance to supplier’s instructions and the estimated consumption from each mouse was circa 25 mL.

The animals were housed in groups of four in cages under a regulated 12h light/12h dark cycle and controlled temperature (22–25 °C) and relative humidity (50%) conditions. Their weight and health status were recorded weekly.

All procedures were in accordance to the current national (Ν. 2015/1992, ΠΔ 56/2013) and European legislation (European guideline 2010/63).

### 4.3. Sample Preparation of Fecal and Urine Samples 

Urine samples, after thawing at room temperature (RT), were vortexed and aliquots of 50 μL were diluted with 150 μL of acetonitrile. Next, the diluted samples were vortexed again, centrifuged at 12,000 *g* for 10 min and the clear supernatant was transferred to LC-MS vials and then to the autosampler at 4 °C for analysis.

Fecal samples were weighed and then homogenized by the addition of saline solution (NaCl 0.9%) in such a volume to obtain a sample weight to saline volume ratio of 1:9 (*w*/*v*) [51]. Homogenation was aided by vortex-mixing and sonication for 10 min. After centrifugation at 12,000 *g* for 20 min, 100 μL of the obtained fecal saline volume was taken and 300 μL of MeOH:H_2_O at a ratio of 1:1 (*v*/*v*) was added. After vortexing and centrifugation, 150 μL of the clear supernatant was collected and evaporated until dryness using a Speed Vac Eppendorf AG (Hamburg, Germany). Prior to the analysis, the dried extract was reconstituted with 150 μL of mixture of MeCN:H_2_O:MeOH at a volume ratio of 70:15:15 (*v*/*v*/*v*).

### 4.4. LC-MS/MS analysis

The analysis was performed by a targeted hydrophilic interaction liquid chromatography tandem mass spectrometry (HILIC-MS/MS) method which has been previously developed from our group [52] for such studies [53,54,55] targeting more than 100 hydrophilic endogenous metabolites. The analysis was performed on an ACQUITY UPLC H-Class chromatography system combined with a Xevo TQD mass spectrometer (Waters Corporation, Millford, MA, USA) operating in both positive and negative mode, with an Acquity BEH Amide Column (Waters Ltd., Elstree, UK). The mobile phase consisted of MeCN:H_2_O, 95:5 (*v*/*v*) and MeCN:H_2_O, 30:70 (*v*/*v*), both containing 10 mM ammonium formate, pH 6.

The samples were analyzed in a randomized order in two separate batches for each specimen and 5 μL was injected into the system. For the quality assessment of the data, a quality control (QC) sample was prepared for urine or fecal extracts according to published protocols [56] and analyzed every 10 samples within the analytical batch.

### 4.5. Data Analysis

The obtained LC-MS/MS data were collected using MassLynx® (Waters, Milford, MA, USA), while peak identification and integration were accomplished by TargetLynx® (v4.1). Exclusion criteria were either the absence of metabolite in ≥20% of the analyzed samples or metabolite relative standard deviation (RSD) >30% in the QC samples. Metabolites that did not follow the above criteria were excluded from the final dataset.

Univariate statistical analysis using a two-tailed t-test with unequal variance algorithm (a threshold of *p*-value was set at 0.05) was performed to check the impact of each metabolite on the tested hypothesis. Multiple multivariate statistical algorithms (VIP (variable importance for the projection) plots, PCA (principal component analysis) and OPLS-DA (orthogonal partial least squares discriminant analysis) in UV scaling were performed by SIMCA 13.0 (Umetrics, Umea, Sweden) for biomarker discovery and metabolite cross-interaction investigation. The validity of the constructed models was evaluated based on permutation plots and CV-ANOVA value. To check and visualize the performance of the potential biomarkers the area under the curve-receiver operating characteristic (AUC-ROC) curve analysis was performed and the results were illustrated using ROC curves and box-plots graphs.

Evaluation of the models was also performed by additional calculation of AUC values from the ROC curve analysis using the YpredCV value of each model. The quality of the models was determined by the goodness of fit in the X (R2X) and Y (R2Y) variables and the predictability based on the fraction correctly predicted in one-seventh cross-validation (Q2YCV). To calculate the area under the ROC curve (AUROC), specificity, sensitivity and accuracy at the optimum cut-off level during the 7-fold cross validation (Y-predcv, predictive Y variables; SIMCA-P+ software) was performed using the GNU R ROCR package. Biochemical interpretation of differentiated metabolites and the investigation of their metabolic origin “pathway annotation” were performed on MetaboAnalyst 4.0 [57,58].

## Figures and Tables

**Table 1:** Data statistics from all constructed OPLS DA models.

Model	Statistics of the Model						Predictive Ability			
	Apred	Aorth	R2Y	R2X	Q2YCV	*p* (CV ANOVA)	Sensitivity	Specificity	Accuracy	AUC
Fecal samples Control vs. Ethanol TP 1	1	2	0.942	0.567	0.942	2.84 × 10^−8^	1	1	100%	1
Fecal samples Control vs. Ethanol TP 1 (Male)	1	2	0.998	0.768	0.967	7.60 × 10^−5^	1	1	100%	1
Fecal samples Control vs. Ethanol TP 1 (Female)	1	2	0.955	0.583	0.836	9.92 × 10^−5^	1	1	100%	1
Fecal samples Control vs. Ethanol TP 2	1	2	0.925	0.481	0.925	3.00 × 10^−4^	1	0.92	96%	0.99
Fecal samples Control vs. Ethanol TP 2 (Male)	1	4	0.982	0.57	0.8	1.00 × 10^−2^	1	1	100%	1
Fecal samples Control vs. Ethanol TP 2 (Female)	-	-	-	-	-	>1	-	-	-	-
Urine samples Control vs. Ethanol TP 1	1	2	0.791	0.671	0.791	9.70 × 10^−2^	0.76	0.88	81%	0.9
Urine samples Control vs. Ethanol TP 1 (Male)	1	2	0.998	0.657	0.796	1.00 × 10^−2^	1	1	100%	1
Urine samples Control vs. Ethanol TP 1 (Female)	-	-	-	-	-	>1	-	-	-	-
Urine samples Control vs. Ethanol TP 2	1	1	0.877	0.625	0.877	4.93 × 10^−6^	1	0.93	96%	0.99
Urine samples Control vs. Ethanol TP 2 (Male)	-	-	-	-	-	>1	-	-	-	-
Urine samples Control vs. Ethanol TP 2 (Female)	1	1	0.822	0.633	0.708	9.00 × 10^−3^	1	1	100%	1

TP, time point; Apred, number of Y-predictive components; Aorth, number of Y-orthogonal components; R2X, explained variance of X; R2Y, explained variance of Y; Q2YCV, predicted variance of Y estimated using cross-validation. R2X and R2Y show how well the model explains the variation in X and Y, respectively. Q2Y represents the quality and predictive power of the model. Sensitivity (specificity) measures the proportion of actual positives (negatives) that are correctly predicted with the model. Accuracy (ACC) is the proportion of true results (both true positives and true negatives) in all results. The area under the curve (AUROC) is equal to the probability that a classifier will rank a randomly chosen positive instance higher than a randomly chosen negative one.

## Supplementary Materials

The following are available online at https://www.mdpi.com/2218-1989/9/10/232/s1, Figure S1: Metabolites detected in urine and fecal samples of control and ethanol treated mice showing the commonly detected metabolites; Figure S2: OPLS DA score plots of the models constructed for male and female mice samples separately; Figure S3: Bar plots of log2 fold change showing the altered metabolites (*p* < 0.05) based on the models constructed separately for male and female mice separately; Table S1: List of metabolites found to be significantly affected in fecal extracts with their trend and metabolic pathway that they are involved in; Table S2: List of metabolites found to be significantly affected in urine with their trend and metabolic pathway that they are involved in.
