# Supplementary material for: Study of Fecal and Urinary Metabolite Perturbations Induced by Chronic Ethanol Treatment in Mice by UHPLC-MS/MS Targeted Profiling
Source: Metabolites. 2019 Oct 16;9(10):232. doi: 10.3390/metabo9100232 (PMC6836053; doi:10.3390/metabo9100232)
Supplement: Supplementary file 1 [file metabolites-09-00232-s001.pdf]

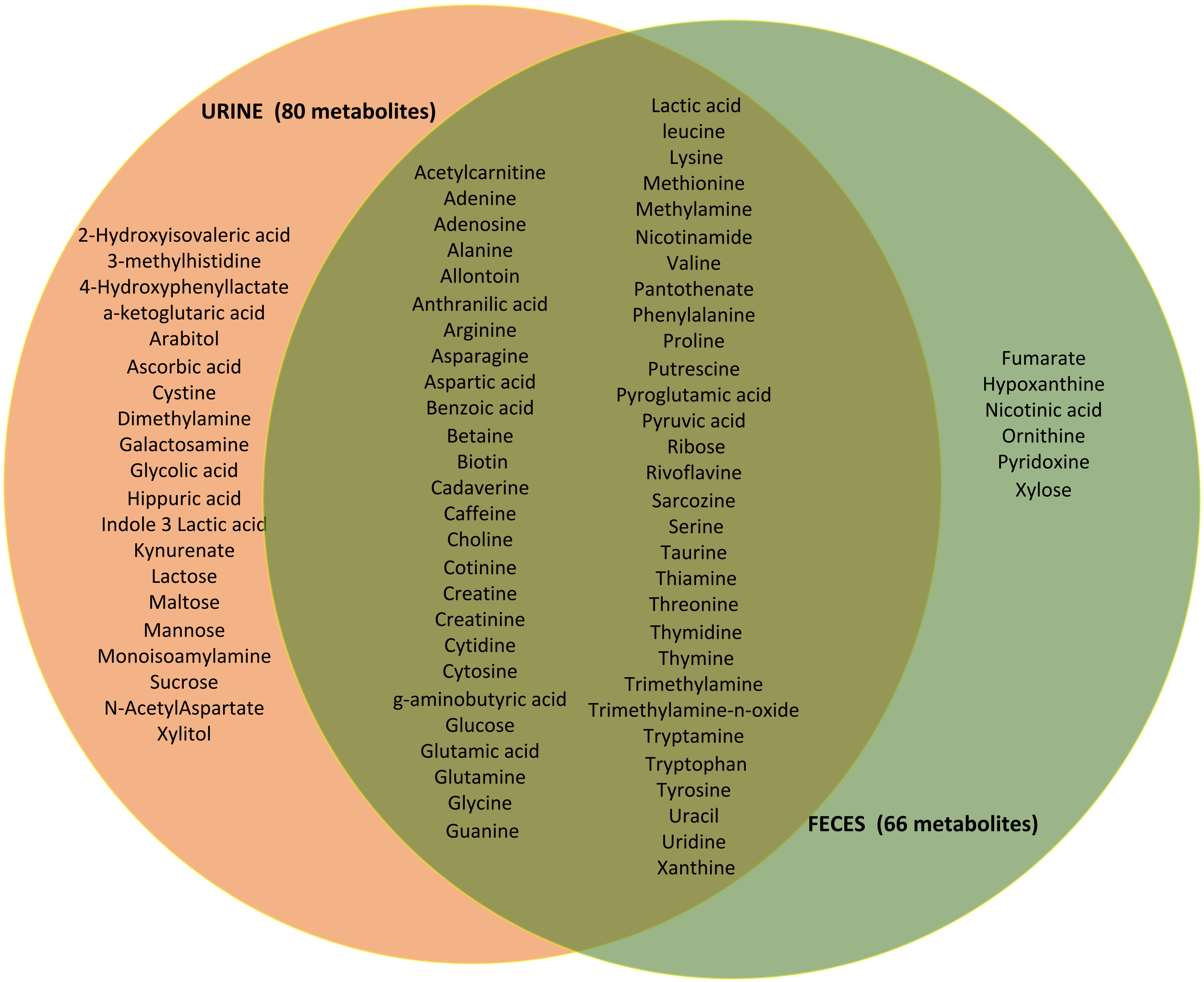


Sup Figure 1.Venn Diagram of detected metabolites in fecal and urine sample

# FECES

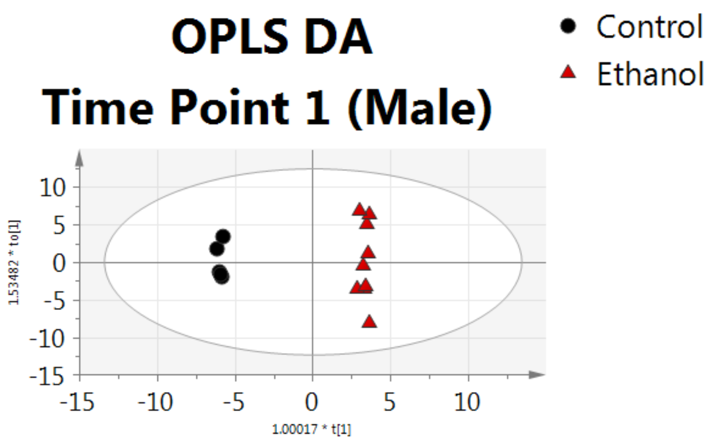


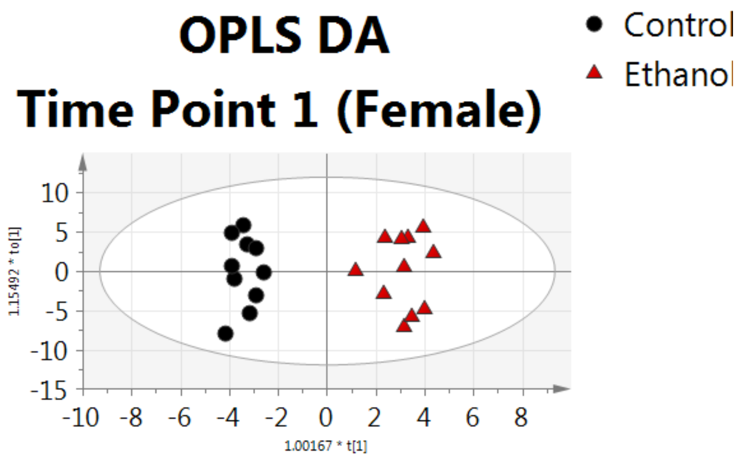


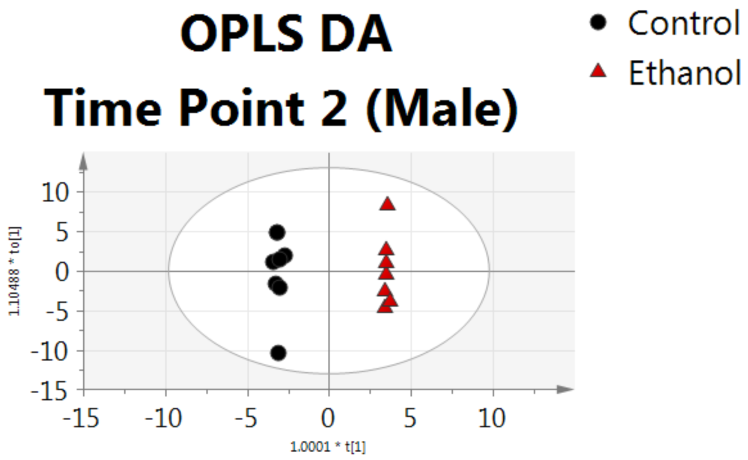


# URINE

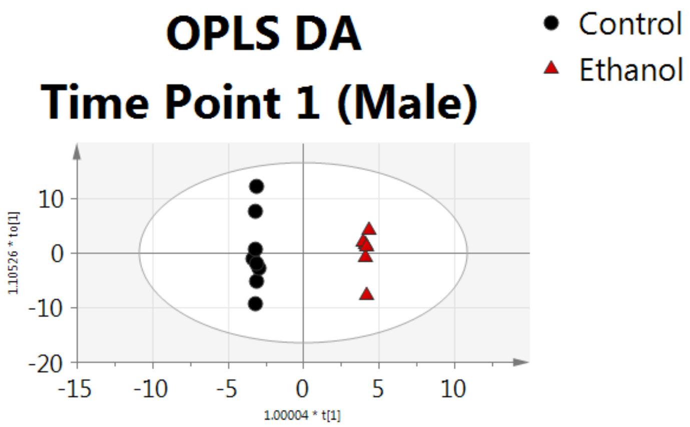


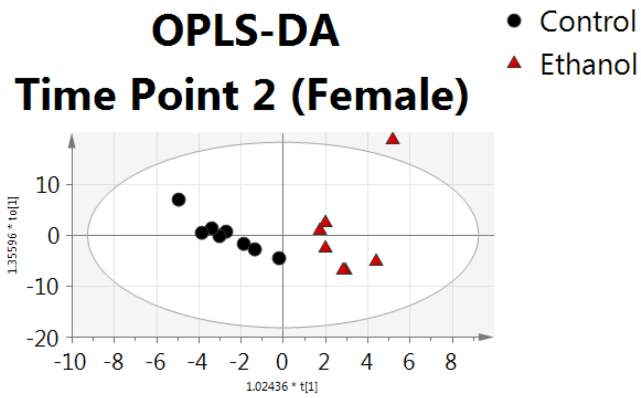


Figure S2: OPLS DA score plots of the models constructed for male and female mice samples separately, (x axis: first principal component t[1], yaxis: the first component to[1])

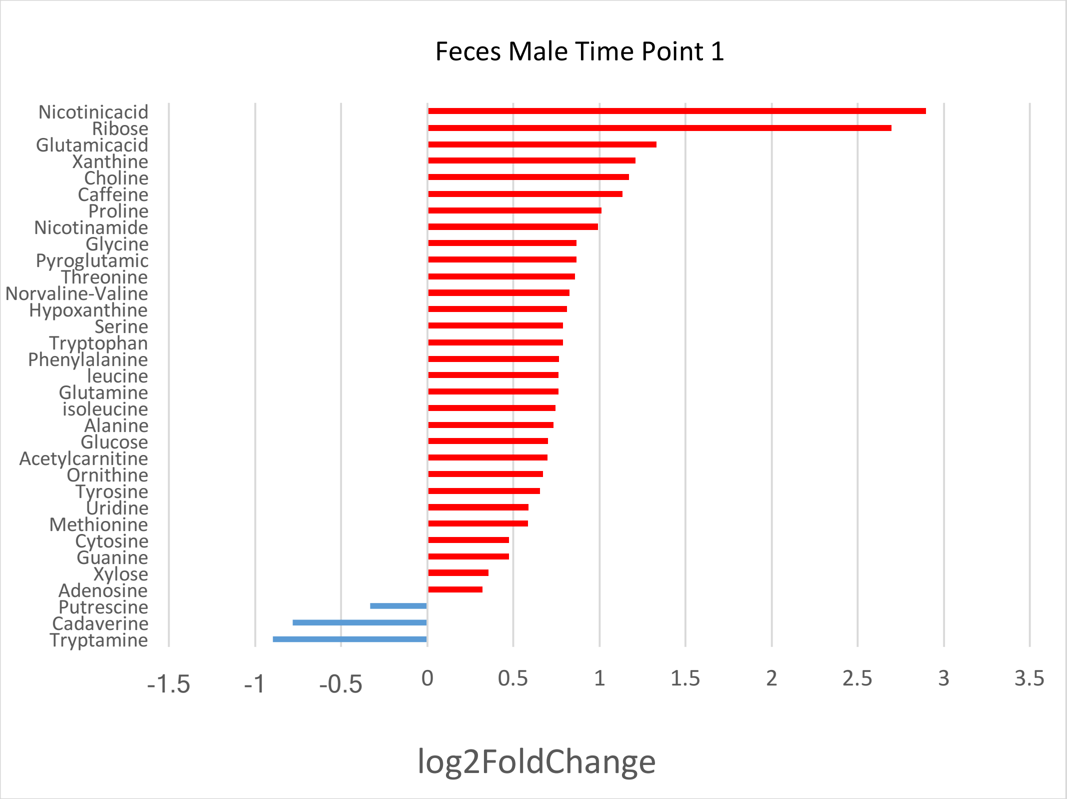
Feces Male Time Point 1
Nicotinicacid
Ribose
Glutamicacid
Xanthine
Choline
Caffeine
Proline
Nicotinamide
Glycine
Pyroglutamic
Threonine
Norvaline-Valine
Hypoxanthine
Serine
Tryptophan
Phenylalanine
leucine
Glutamine
isoleucine
Alanine
Glucose
Acetylcarnitine
Ornithine
Tyrosine
Uridine
Methionine
Cytosine
Guanine
Xylose
Adenosine
Putrescine
Cadaverine
Tryptamine
-1.5
-1
-0.5
0
0.5
1
1.5
2
2.5
3
3.5
log2FoldChange

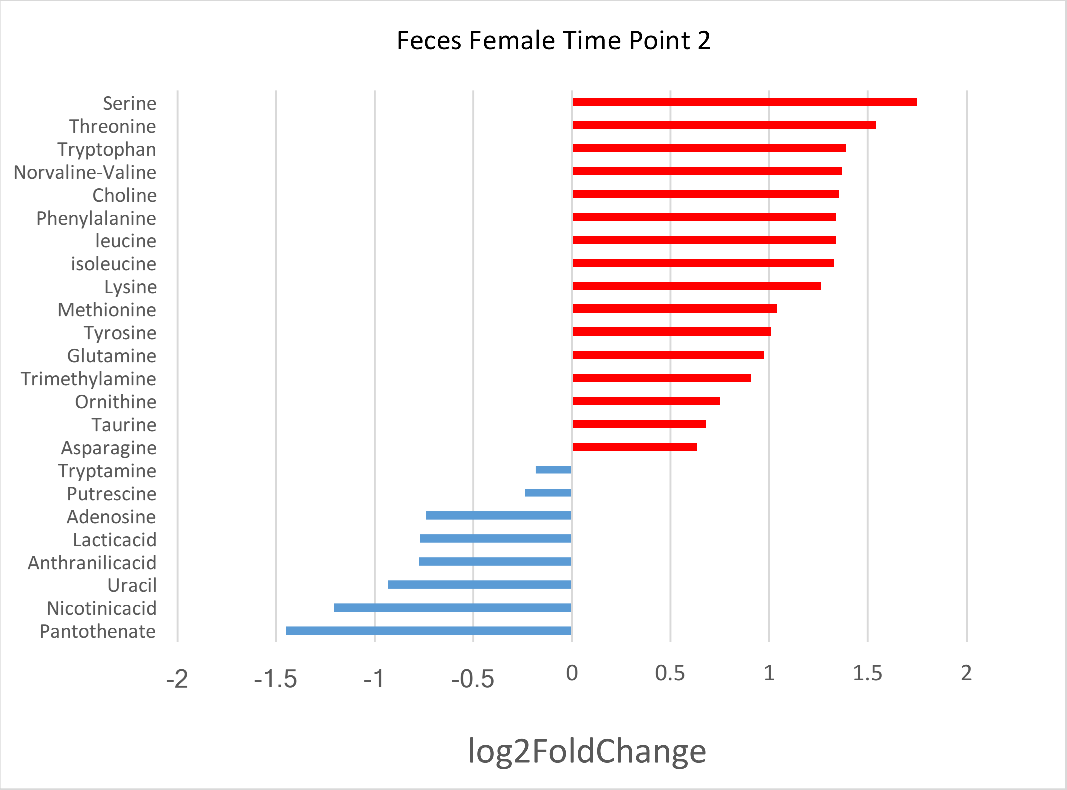
Feces Female Time Point 2
Serine
Threonine
Tryptophan
Norvaline-Valine
Choline
Phenylalanine
leucine
isoleucine
Lysine
Methionine
Tyrosine
Glutamine
Trimethylamine
Ornithine
Taurine
Asparagine
Tryptamine
Putrescine
Adenosine
Lacticacid
Anthranilicacid
Uracil
Nicotinicacid
Pantothenate
-2
-1.5
-1
-0.5
0
0.5
1
1.5
2
log2FoldChange

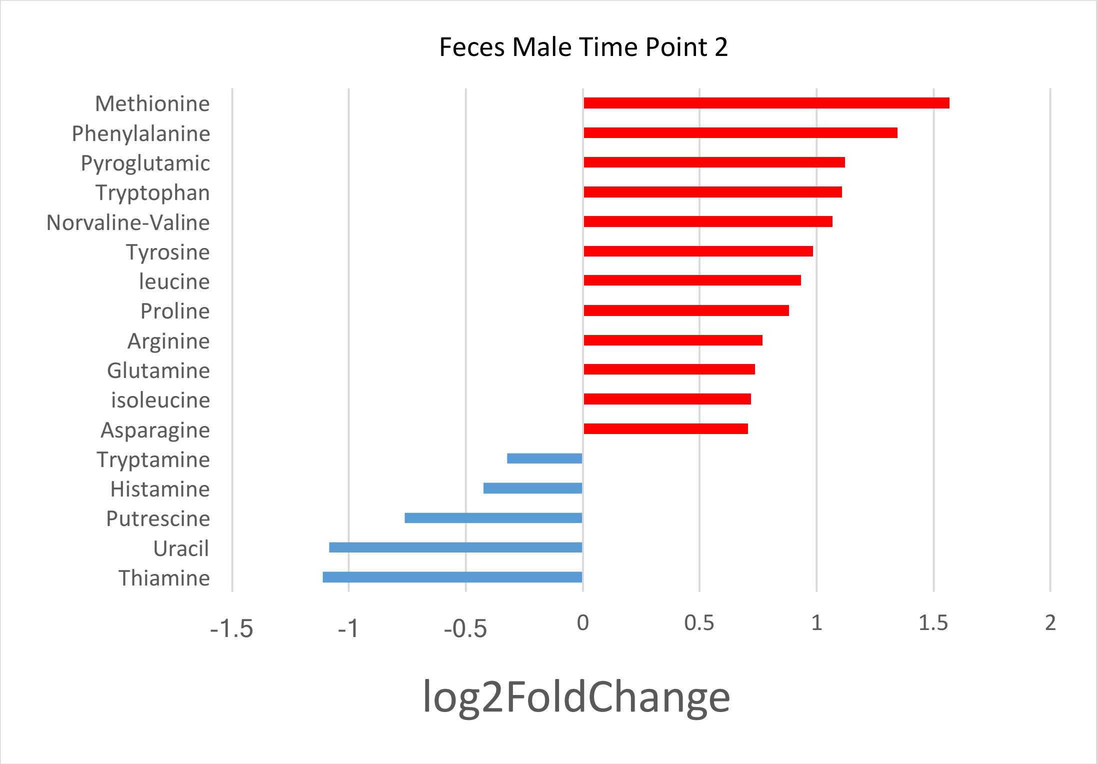
Feces Male Time Point 2
Methionine
Phenylalanine
Pyroglutamic
Tryptophan
Norvaline-Valine
Tyrosine
leucine
Proline
Arginine
Glutamine
isoleucine
Asparagine
Tryptamine
Histamine
Putrescine
Uracil
Thiamine
-1.5
-1
-0.5
0
0.5
1
1.5
2
log2FoldChange

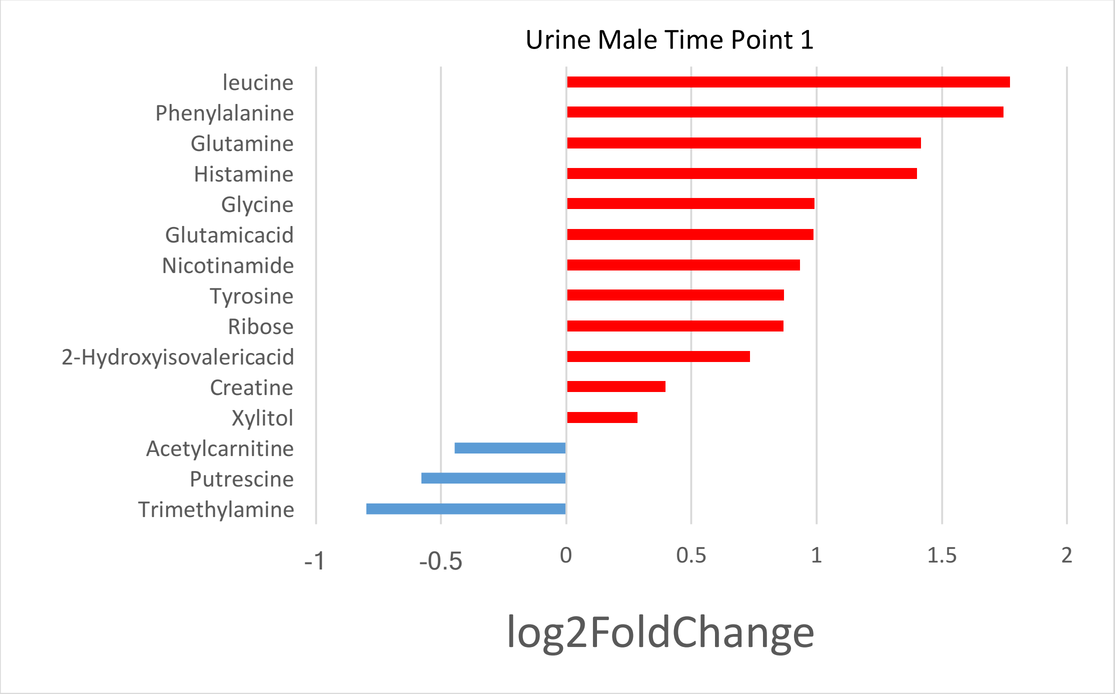
Urine Male Time Point 1
leucine
Phenylalanine
Glutamine
Histamine
Glycine
Glutamicacid
Nicotinamide
Tyrosine
Ribose
2-Hydroxyisovalericacid
Creatine
Xylitol
Acetylcarnitine
Putrescine
Trimethylamine
-1
-0.5
0
0.5
1
1.5
2
log2FoldChange

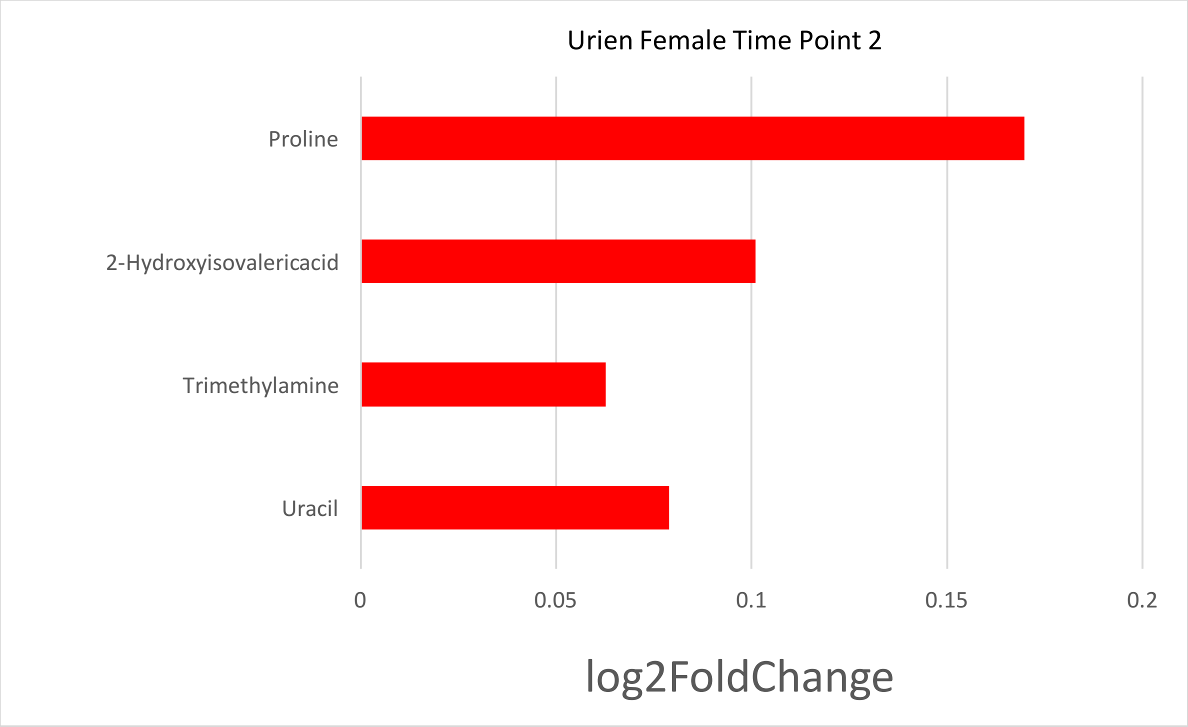


Figure S3: Bar plots of log2 Fold Change showing the altered metabolites (*p<0.05*) based on the models constructed for male and female mice separately (x axis: lg2fold change value, y axis: compound name)

Table S1: Differentiated metabolites from the analyzed fecal samples, their related biochemical pathway and their trend of groups and time points are presented.

| | | **1st Timepoint** | **2nd Timepoint** |
|---|---|---|---|
| | **Potential Related Biochemical Pathways** | Ethanol Group | Ethanol Group |
| Choline | Glycine, serine and threonine metabolism | | ↑ |
| Cytosine | Pyrimidine metabolism | | ↑ |
| **D-Glucose** | Glycolysis / Gluconeogenesis, Pentose phosphate pathway | ↑ | ↑ |
| D-Xylose | Pentose and glucuronate interconversions | | ↑ |
| Glycine | Glycine, serine and threonine metabolism, Purine metabolism | | ↑ |
| Hypoxanthine | Purine metabolism | | ↑ |
| **L-Acetylcarnitine** | Fatty acid degradation | ↓ | ↑ |
| L-Alanine | Alanine, aspartate and glutamate metabolism | | ↑ |
| L-Arginine | Arginine biosynthesis, Arginine and proline metabolism | ↑ | |
| **L-Asparagine** | Alanine, aspartate and glutamate metabolism | ↑ | ↑ |
| L-Aspartic acid | Alanine, aspartate and glutamate metabolism, Arginine biosynthesis | | ↑ |
| **L-Glutamine** | Arginine biosynthesis, Purine metabolism, Pyrimidine metabolism, Alanine, aspartate and glutamate metabolism /D-Glutamine and D-glutamate metabolism | ↑ | ↑ |
| **L-Isoleucine / L-Leucine** | Valine, leucine and isoleucine degradation/ biosynthesis | ↑ | ↑ |
| L-Lactic acid | Glycolysis / Gluconeogenesis, Fructose and mannose metabolism, Pyruvate metabolism | ↓ | |

| | | | |
|---|---|---|---|
| **L-Lysine** | Lysine biosynthesis/ degradation | ↑ | ↑ |
| L-Methionine | Cysteine and methionine metabolism | | ↑ |
| **L-Phenylalanine** | Phenylalanine metabolism, Phenylalanine, tyrosine and tryptophan biosynthesis | ↑ | ↑ |
| **L-Proline** | Arginine and proline metabolism | ↑ | ↑ |
| L-Serine | Glycine, serine and threonine metabolism | | ↑ |
| L-Threonine | Glycine, serine and threonine metabolism | | ↑ |
| **L-Tryptophan** | Glycine, serine and threonine metabolism, Tryptophan metabolism, Phenylalanine, tyrosine and tryptophan biosynthesis | ↑ | ↑ |
| **L-Tyrosine** | Tyrosine metabolism, Phenylalanine metabolism, Phenylalanine, tyrosine and tryptophan biosynthesis | ↑ | ↑ |
| **L-Valine** | Valine, leucine and isoleucine degradation/ biosynthesis | ↑ | ↑ |
| Methylamine | Methane metabolism | | ↑ |
| Niacinamide | Nicotinate and nicotinamide metabolism | | ↑ |
| Nicotinic acid | Nicotinate and nicotinamide metabolism | ↓ | |
| **Ornithine** | Arginine biosynthesis, Arginine and proline metabolism | ↑ | ↑ |
| **Putrescine** | Arginine and proline metabolism | ↓ | ↑ |
| Pyroglutamic acid | Glutathione metabolism | | ↑ |
| Taurine | Taurine and hypotaurine metabolism, Primary bile acid biosynthesis, | | ↑ |
| **Thiamine** | Thiamine metabolism | ↓ | ↓ |
| Trimethylamine | Methane metabolism | | ↑ |
| **Tryptamine** | Tryptophan metabolism | ↓ | ↑ |
| **Uracil** | Pyrimidine metabolism | ↓ | ↓ |
| Uridine | Pyrimidine metabolism | ↑ | |

Table S2: Differentiated metabolites from the analyzed urine samples, their related biochemical pathway and their trend of groups and time points are presented.

| | | **1st Timepoint** | **2nd Timepoint** |
|---|---|---|---|
| | **Potential Related Biochemical Pathways** | Ethanol Group | Ethanol Group |
| **(S)-3-Hydroxyisobutyric acid** | Valine, leucine and isoleucine degradation | ↑ | ↑ |
| Cadaverine | Lysine degradation, Glutathione metabolism | ↓ | |
| D-Maltose | Starch and sucrose metabolism | ↑ | |
| **D-Ribose** | Pentose phosphate pathway | ↑ | ↑ |
| Glycine | Glycine, serine and threonine metabolism, Purine metabolism | | ↑ |
| Guanine | Purine metabolism | ↑ | |
| **Hydroxyphenyllactic acid** | Tyrosine metabolism | ↑ | ↑ |
| **Indolelactic acid** | Tryptophan metabolism | ↑ | ↑ |
| L-Acetylcarnitine | Fatty acid degradation | | ↑ |
| L-Cystine | Cysteine and methionine metabolism | ↑ | |
| L-Glutamic acid | Arginine biosynthesis, Alanine, aspartate and glutamate metabolism, Arginine and proline metabolism | | ↑ |
| L-Glutamine | Arginine biosynthesis, Purine metabolism, Pyrimidine metabolism, Alanine, aspartate and glutamate metabolism<br>/D-Glutamine and D-glutamate metabolism | | ↑ |
| **L-Isoleucine/L-isoleucine** | Valine, leucine and isoleucine degradation/ biosynthesis | ↑ | ↑ |
| Niacinamide | Nicotinate and nicotinamide metabolism | | ↑ |

| | | | |
|---|---|---|---|
| Putrescine | Arginine and proline metabolism | | ↓ |
| Pyroglutamic acid | Glutathione metabolism | | ↑ |
| **Trimethylamine** | Methane metabolism | ↓ | ↓ |
| Trimethylamine N-oxide | Methane metabolism | ↑ | |
| Uracil | Pyrimidine metabolism | ↓ | |
